# Analysis of the effects of simplifications on the state of loads in a centrifugal compressor

**DOI:** 10.1038/s41598-022-20753-z

**Published:** 2022-10-07

**Authors:** Arkadiusz Bednarz, Jarosław Sęp, Rafał Bartłomowicz, Justin Jaworski, Augustyn Wójcik

**Affiliations:** 1 Faculty of Mechanical Engineering and Aeronautics, Rzeszow University of Technology, 35-959 Rzeszów, Poland; 2 Department of Mechanical Engineering and Mechanics, Lehigh University, Bethlehem, PA 18015 USA; 3 Wytwórnia Urzadzeń Chłodniczych “PZL-Debica” S.A., 39-200 Debica, Poland

**Keywords:** Aerospace engineering, Mechanical engineering, Computational science

## Abstract

This study aims to quantify and assess qualitatively the impact of modeling simplifications used to represent inertial and aerodynamic loads on the stresses and structural deformations of a centrifugal compressor in operation. The research object is the compressor of the high-pressure line of the DGEN 380 bypass turbine engine. Based on the virtual dynamometer WESTT CS/BV, the gas-dynamic parameters at the entrance to the centrifugal compressor and after the stage are determined. These values were used as initial parameters for numerical flow analysis. As part of the numerical strength analyses, a series of several load configurations were carried out: spin only, spin and inlet pressure normally applied on the working surface of the rotor blade, spin and outlet pressure normally applied on the working surface of the rotor blade, and one-way fluid–structure interaction analysis taking into account the aerodynamic loads with and without spinning. Based on the simulations, the level of similarity of a given load configuration with the last analysis, adopted as the reference, was determined. It was observed that in terms of the stress state, the rotational analysis taking into account the pressure on both sides of the blade gives satisfactory results, but the strain values are overestimated. The results obtained and the method of evaluation of compressor results can be used in research in the area of aviation, automotive, and refrigeration industries.

## Introduction

Compressors are one of the most critical elements of gas turbine engines^1^ and strongly influence the overall system design and operation requirements^2^. These requirements arose as a result of the complex load and operating conditions of the rotating machines. Operating conditions require robustness of the compressor to both relatively high rotational speeds and aerodynamic loads^3–5^, and to the risk of damage caused by external factors (i.e., foreign object damage^6^).

At the design stage for centrifugal compressors, analytical calculations and numerical analyses are typically carried out with the aim to determine the state of loads as accurately as possible to validate the stiffness and strength conditions set at the stage of preliminary assumptions. The design of a compressor or turbine itself begins with determining the assumptions of the work, preliminary aerodynamic calculations, geometric calculations, and numerical verification. This last action, apart from later experimental studies, is the key part of the design process: the results of numerical tests were the basis for the decision to approve the geometry and subsequent certification of the product. Numerical fluid and structural analyses were used to determine working conditions as well as the state of loads and deformations of the tested object^7,8^. The most realistic and accurate results are obtained through the cooperation of both tools: fluid–structure interactions (FSI) analyses to take into account the simulated distribution of aerodynamic loads^9–12^, which appear at the boundaries of the flow domain, in the numerical structural analysis^13–15^. The aerodynamic loads obtained by the Finite Volume Method (FVM) simulation (based on the Reynolds-averaged Navier–Stokes equations, or RANS) are currently considered to be closest to the real load condition^16–20^, obtained in a relatively short time.

A well-prepared flow analysis requires scrupulous determination of the operating conditions of a given flow machine^21^, as well as the careful preparation of the computational fluid domain of the compressor flow channel. As part of the operating conditions, the boundary conditions and turbulence models should be distinguished. A similar approach is necessary when modeling structural analysis^22,23^.

Unfortunately, due to the lack of equipment, technology, and knowledge, a simplified approach to numerical analysis is used and is limited only to structural calculations^24–26^. In such cases, the aerodynamic loads were neglected (considered negligible, or an additional safety factor has been adopted) or are incorporated via simplified modeling^27,28^.

In this paper, the authors will evaluate the impact of the method of modeling aerodynamic loads and computational simplifications on the state of loads and deformations of the centrifugal compressor. For this purpose, the authors use the results from the DGEN 380 turbine engine test bench and, more specifically, the data on gas-dynamic parameters upstream and downstream of the high-pressure line centrifugal compressor stage. The mentioned results are used to build and validate the flow analysis in the compressor stage. Then, this analysis is coupled with the computational structural analysis, and the state of loads and deformations of the compressor blades is determined. For comparative purposes, analyses will be carried out taking into account various simplified methods of modeling aerodynamic loads in the structural analysis, and the finite element analysis for the spinning itself is also performed. The differences obtained in the results and locations of the maximum values of the measured quantities will be used for the quantitative and qualitative assessment of the simplifications in aerodynamic loads modeling. These simplifications can be used to inform the modeling for design of centrifugal compressors used in transportation and industrial applications, including automotive, energy, and refrigeration applications. The possibility of simplified modeling of aerodynamic loads as well as their correct assessment and knowledge of the error allows to speed up the process of designing a new compressor and reduces costs in the absence of a solver or specialist in the field of computational fluid dynamics (CFD) analysis.

## The object of study

The research object is the centrifugal compressor of the high-pressure line of the DGEN 380 turbine engine. The DGEN 380 engine was developed in 1996 and was produced by Price Induction. This engine is a turbofan engine with a high bypass ratio of 7.6^26,29,30^. The engine has been designed for a mass flow of 13 kg/s and a maximum thrust of 255 daN, and the temperature of the exhaust gas flow before the turbine reaches the value of 1178 K.

**Figure 1:**
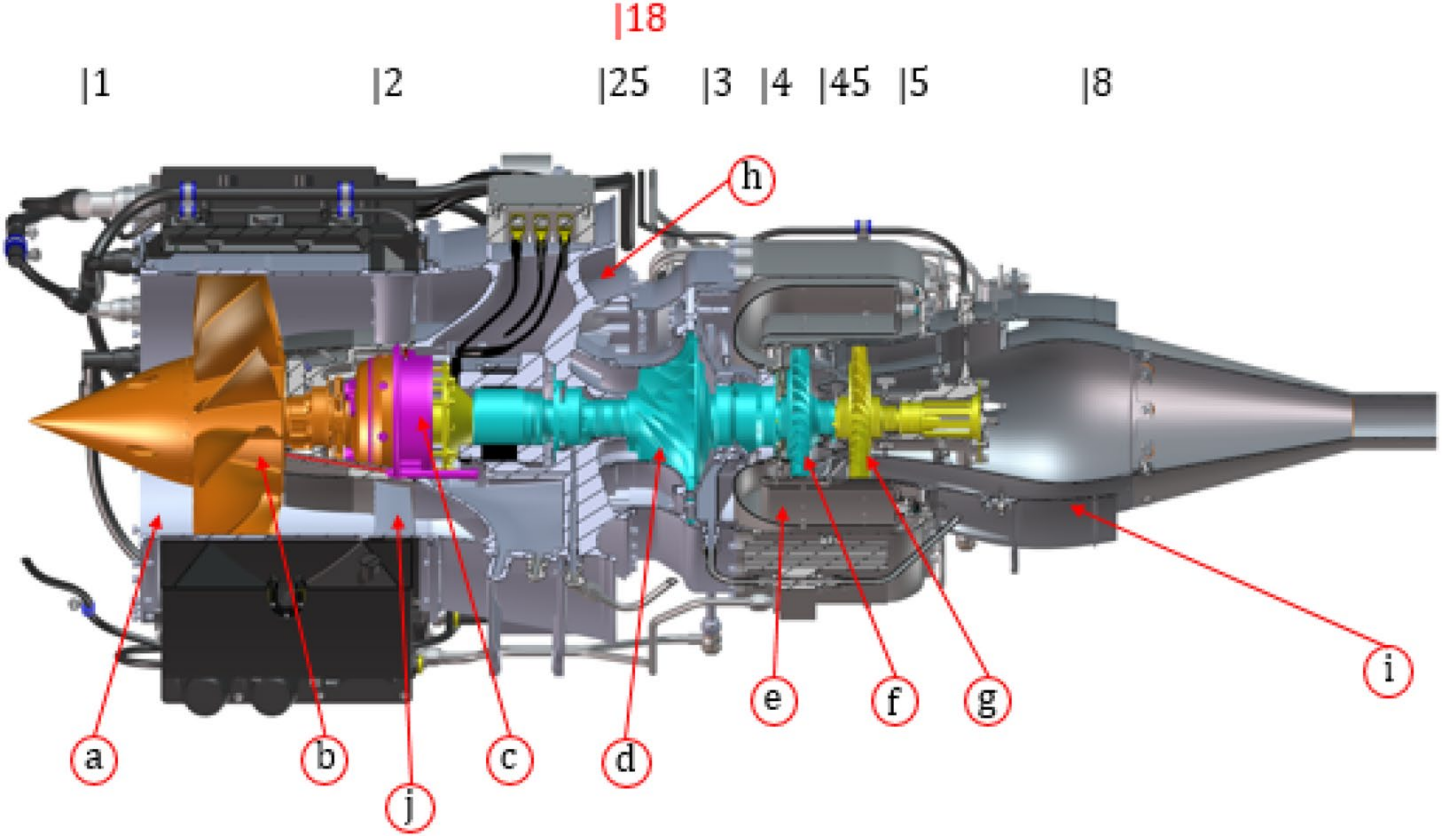
Cutaway illustration of DGEN 380^30,32^ engine: (**a**) engine inlet, (**b**) fan, (**c**) gearbox, (**d**) high-pressure compressor, (**e**) combustion chamber, (**f**) high-pressure turbine, (**g**) low-pressure turbine, (**h**) core nozzle, (**i**) fan nozzle, (**j**) stator.

This engine, and more precisely its virtual version^30^, was built in the virtual WESTT CS/BV dynamometer^26,30,31^. This dynamometer was a simulation stand in which, depending on the input parameters, it is possible to monitor engine thrust, fuel consumption, rotational speeds of specific rotors, as well as gas-dynamic parameters in all key sections of the engine (Fig. 1). The view of the engine was prepared thanks to documentation provided to the WESTT CS/BV by the Price Induction^30^ and Catia Composer^32^. These data (temperatures, pressures, rotational speeds, and velocity triangles) form the basis to create the boundary conditions and validates the results of the numerical flow analysis in this work. The selected operating conditions correspond to the data from the real engine.

For flow tests, based on the literature review^31,33^ and certification guidelines^2^, the conditions of motor power were selected for cruising speed at sea level ($\hbox {H}=0$ m above sea level). The data obtained on temperatures and pressures were summarized in Table 1.

**Table 1:** Parameters of the DGEN 380 engine (in sections of the engine).

Name of parameter	Value
Inlet static temperature—T$_{1}$	288.15 K
Inlet static pressure—p$_{1}$	101.325 kPa
Total temperature—T$_{2}^{*}$	288.15 K
Total pressure—p$_{2}^{*}$	101.2 kPa
Total temperature—T$_{25}^{*}$	304.5 K
Total pressure—p$_{25}^{*}$	116.3 kPa
Total temperature—T$_{3}^{*}$	512.8 K
Total pressure—p$_{3}^{*}$	534.5 kPa
Total temperature—T$_{4}^{*}$	1177.44 K
Total pressure—p$_{4}^{*}$	510.2 kPa
Total temperature—T$_{45}^{*}$	990.2 K
Total pressure—p$_{45}^{*}$	227 kPa
Total temperature—T$_{5}^{*}$	867 K
Total pressure—p$_{5}^{*}$	122.2 kPa
Total temperature—T$_{8}^{*}$	867 K
Total pressure—p$_{8}^{*}$	120 kPa
Total temperature—T$_{18}^{*}$	306.8 K
Total pressure—p$_{18}^{*}$	120.8 kPa
Fuel mass flow—m$_{\mathrm{f}}$	0.0314 kg/s
Specific fuel consumption (SFC)	0.45 kg/(h*daN)
Thrust—F	249.1 daN

**Figure 2:**
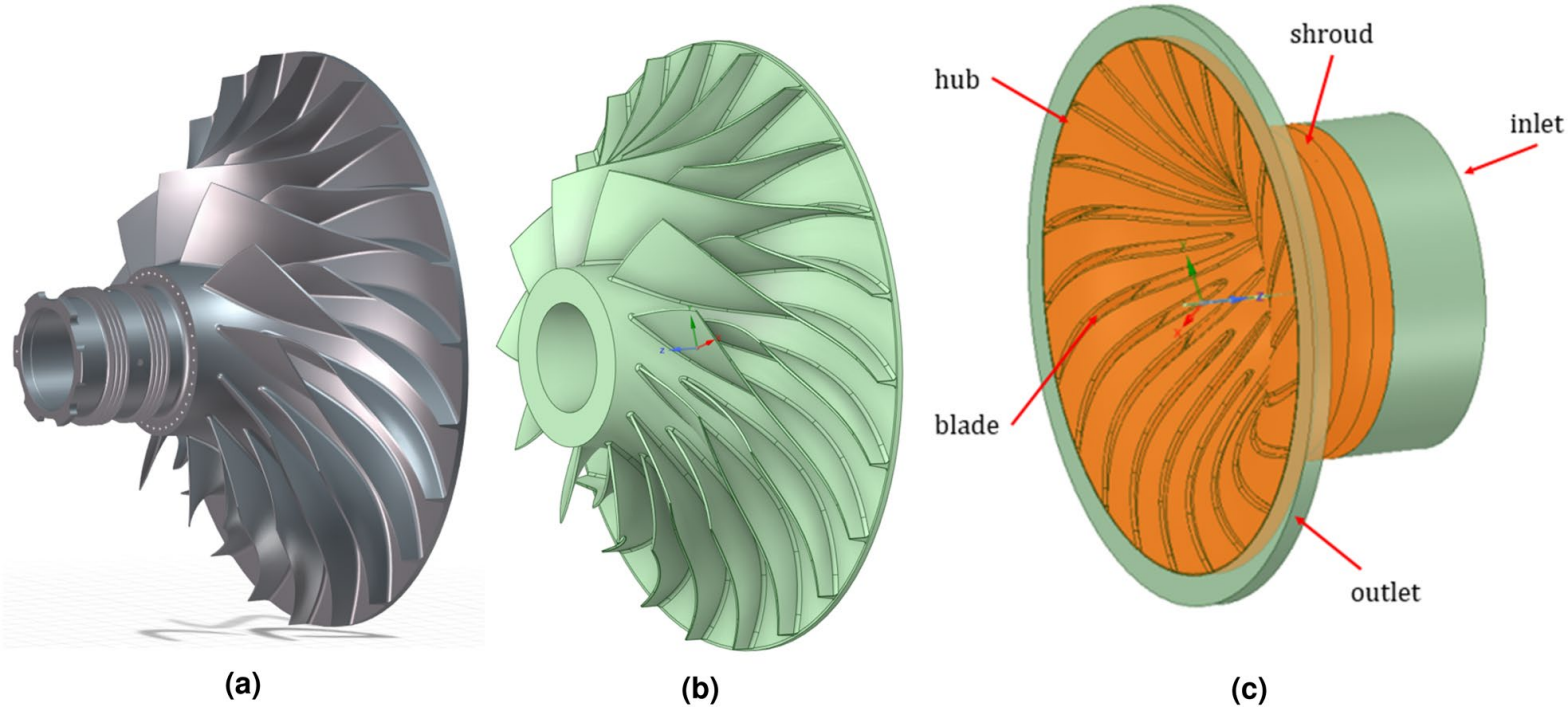
View of centrifugal compressor of DGEN 380: (**a**) geometrical model from the virtual dynamometer^30^, (**b**) simplified geometrical model for FEM analysis (Ansys Mechanical^34^ and (**c**) fluid domain (Ansys CFX^35^).

Additionally, for the flow analysis, the geometry of the centrifugal compressor from the high-pressure line, Fig. 2a, was reconstructed. The outer diameter of the compressor is 200 mm. The compressor design includes 11 blades and 11 splitters. The geometric model of the compressor rotor was created using a reverse-engineering methodology, i.e. by recreating the geometry using a 3D scan and mathematical mapping of the blade surface, Fig. 2b. This compressor spins at 51,410 RPM under cruise conditions. According to the engine documentation, about 1.78 kg/s flows through the inner passage, i.e. about 0.162 kg/s through one inter-vane zone of the rotor of the centrifugal compressor.

## Numerical flow analysis

The geometrical model of the rotor (Fig. 2b) was used to build the surrounding fluid domain model. It is necessary to take into account the clearance (0.2 mm) between the tip of the vane and the outer casing and to take into account the compressor inlet and outlet. The prepared fluid domain model with named specific surfaces defining the boundary conditions is presented in Fig. 2c.

**Table 2:** Comparison of CFD and experimental data from the test stand.

Parameter	Dynamometer value	CFD average result	Error, %
Total pressure p$_{3}^{*}$, kPa	542.98	534.9	1.51
Temperature T$_{3}^{*}$, K	489.12	510.1	4.11
Absolute velocity, m/s	390.49	406	3.82

The flow analysis itself is performed using Ansys CFX software^35^. At the inlet, the speed, static pressure, and temperature are set to the values from the virtual dynamometer data in Table 1. On the shroud surface representing the compressor housing, stationary conditions were assumed, while on the surfaces of the blades and the hub, rotational conditions were assumed. At the outlet, an outflow condition (value of the mass flow rate) was adopted, in which the values of the damming pressure, temperature, and speed are verified.

The flow analysis uses the RANS fluid equations with the k-$\omega$ SST turbulence model^21–23,36^. The analysis was performed as a steady analysis (i.e., temporal changes in the domain not taken into account) with the pseudo-transient option. The working medium was the air with parameters corresponding to the conditions at sea level. The fluid domain itself was built based on tetrahedrons with 4-nodes (TET-4) elements with an average dimension of 5 mm. On the external surfaces of the flow domain, the condition of creating inflation layers was assumed. The generation of 12 inflation layers with the height of the first layer at the level of 0.02 mm was assumed. As a result of the creation of the discussed mesh, a domain consisting of nearly 6.5 million finite volumes was obtained with a skewness value at the level of 0.28 and an orthogonal quality value of 0.71. According to the literature^37^, such mesh parameters are considered very good.

**Figure 3:**
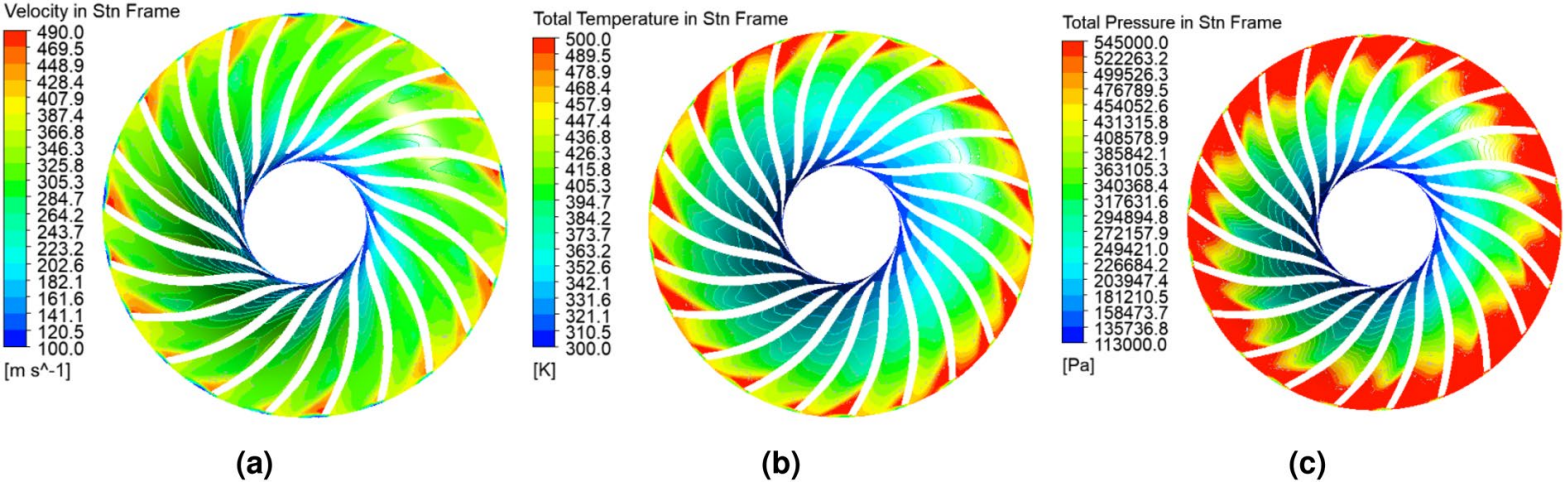
Distribution of (**a**) total velocity, (**b**) total temperature and (**c**) total pressure on the surface halfway through the flow channel (Ansys CFX^35^ visualization).

The results of the numerical flow analysis will be the values of temperature, pressure, and velocity at the outlet from the domain presented in Table 2, and the distributions of these values (velocity, Fig. 3a, total temperature—Fig. 3b, and total pressure—Fig. 3c) on the surface halfway through the flow channel (50% of the blade height). This analysis predicts a maximum flow speed in the stationary reference frame at the compressor exit of about 490 m/s (Fig. 3a). A similar distribution and location of maximum values up to 500 K (Fig. 3b) are observed for the temperature distribution. In the case of the total pressure distribution (Fig. 3c), its maximum values are observed at the outlet of the channel.

The values at the outlet (averaged for the entire area) of the domain are summarized in Table 2 and are compared with the results obtained from the virtual test bench (Table 1). Based on the flow analysis, it is estimated that the total pressure behind the compressor was 534.9 kPa, which gives an error of 1.51% relative to the result of the virtual dynamometer. The highest error (4.11%) is associated with the stagnation temperature, which in the flow analysis was 510.1 K. The reason for such an error is likely the lack of consideration of heat conduction through the housing walls and from the rotor to the shaft. The velocity at the exit from the domain predicted by numerical analysis was 406 m/s. This value was about 15 m/s higher than the value obtained from the virtual dynamometer. Therefore, the CFD analysis is validated by the general close agreement of outflow metrics with the virtual dynamometer.

**Figure 4:**
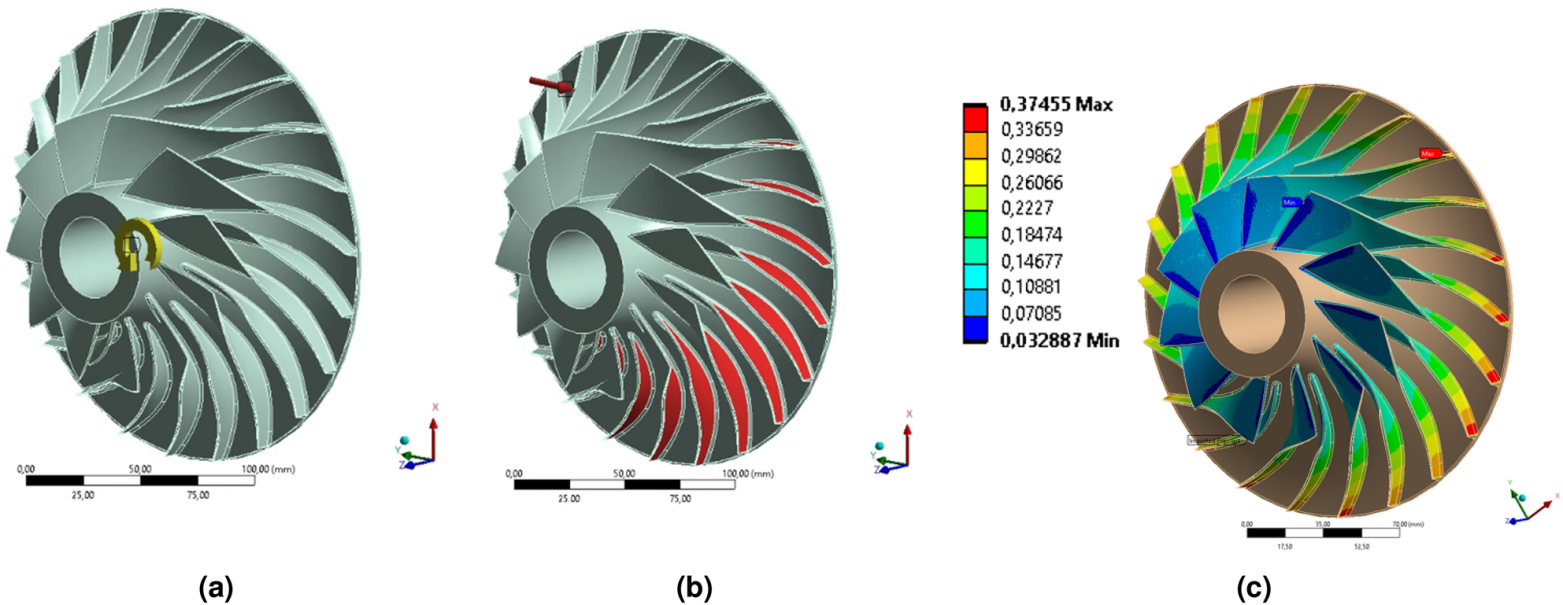
Definition of the boundary conditions: (**a**) rotational velocity, (**b**) pressure side, and (**c**) total pressure (MPa) from CFD analysis (Ansys Mechanical^34^ visualization).

The last result generated by numerical flow analysis was the pressure distribution on the working surfaces of the rotor (blades and hub). This distribution (Fig. 4c) is imposed on the numerical structural analysis to conduct the FSI simulation.

## Numerical structural analysis

To perform the structural analysis, it was necessary to prepare a material model, determine the load boundary conditions, and prepare a discrete model needed for the finite element analysis (FEM). FEM analyzes were made on the basis of commercial Ansys Mechanical software^34^. The geometrical model is the rotor of the DGEN 380 centrifugal compressor (Fig. 2b) made of 7075-T6 aluminum alloy. This alloy was characterized by the following chemical composition (excluding Al): 1.6% Cu, 2.5% Mg, 0.23% Cr, and 5.6% Zn^38^. The properties of alloy 7075-T6 are summarized in Table 3.

Since the main goal of the numerical tests carried out is to determine the distribution of stresses ($\sigma$ $_{\mathrm{eqv}}$—equivalent von Mises) and deformations (u$_{\mathrm{tot}}$—total; u$_{\mathrm{cir}}$—circumferential; u$_{\mathrm{rad}}$—radial) in the rotor blades, a constant rotational speed of 51,410 RPM is adopted for the entire object, corresponding to the rotational speed adopted for the flow analysis. In addition, the rotor pressure (inner) surface of the bore (which works with the shaft under real conditions) was blocked.

**Table 3:** Mechanical data of the 7075-T6 alloy^38–40^.

Mechanical data	Value
Density	2.81 g/cm$^3$
Elastic modulus	70 GPa
Poisson ratio	0.3
Kirchhoff modulus	27 GPa
Ultimate tensile strength (UTS)	570 MPa
Tensile yield strength	505 MPa
Elongation (for UTS)	0.1

The structural analysis model is composed of nearly 12 million tetrahedral elements (TET-10) with 10 nodes and square shape functions^41–43^. This type of finite element permits a satisfactory representation of the roundings and curves contained in the rotor geometry and also allowed for the elimination of errors related to the use of deformed elements (pyramids and wedges). Additionally, the compaction of discrete elements in the blade leaves and rounding zones were introduced. This action was taken to improve the quality of the numerical analysis results and to ensure the condition of a minimum of 3 elements along with the thickness of the blade^44,45^.

As part of the structural calculations, 4 types of loads were selected that will be used in the subsequent configurations of numerical analyses: (1) load due to mass forces, from spinning (Fig. 4a); normal pressure applied to the (2) pressure (Fig. 4b) or (3) suction surface of the blade; and (iv) aerodynamic loads from the flow analysis (Fig. 4c). Based on the list of types of loads, 6 analyses can be distinguished, the details of which were summarized in Table 4.

The analysis marked as No. 1 (Table 4) was characterized only by inertial loads resulting from the rotational speed equal to 51,410 RPM—Fig. 4a. Analysis No. 2 takes into account the centrifugation, as well as the pressure of 534.5 kPa (equal to the pressure after compression—Table 1, value p$_{3}^{*}$) on the pressure surface of the blade (Fig. 4b). Analysis No. 3 takes into account the aforementioned loads (spin and pressure on the inner surface of the feather) as well as the pressure on the outer (suction) surface equal to 116 kPa (value from Table 1, p$_{25}^{*}$). The next analysis (No. 4) takes into account the increased value of the normal pressure on the external side equal to the arithmetic mean value of p$_{25}^{*}$ and p$_{3}^{*}$ (325.2 kPa).

The last two analyses performed used the results of the numerical flow analysis to prescribe the surface pressures on the compressor blades. Taking into account the pressure distribution from the CFD analysis (Fig. 4c) allows the mapping of the actual operating conditions of the rotor. Analysis No.5 takes into account the aerodynamic loads but does not take into account the spinning. The last analysis (No. 6) takes into account both the aerodynamic loads from the flow analysis and the mass forces associated with spinning (full FSI analysis).

**Table 4:** Results of the FE analysis.

Analyze number	Rotation	Inner wall pressure (534.5 kPa)	Outer wall pressure	CFD loads	Max u$_{\mathrm{tot}}$, mm	Max u$_{\mathrm{cir}}$, mm	Max u$_{\mathrm{rad}}$, mm	Max $\sigma$ $_{\mathrm{eqv}}$, MPa
1	Yes	No	No	No	0.721	0.503	0.151	405.41
2	Yes	Yes	No	No	0.362	0.137	0.146	387.5
3	Yes	Yes	116 kPa	No	0.363	0.158	0.147	391.36
4	Yes	Yes	325.2 kPa	No	0.441	0.317	0.149	398.34
5	No	No	No	Yes	0.064	0.00063	− 0.0421	12.1
6	Yes	No	No	Yes	0.684	0.478	0.151	350.66

**Figure 5:**
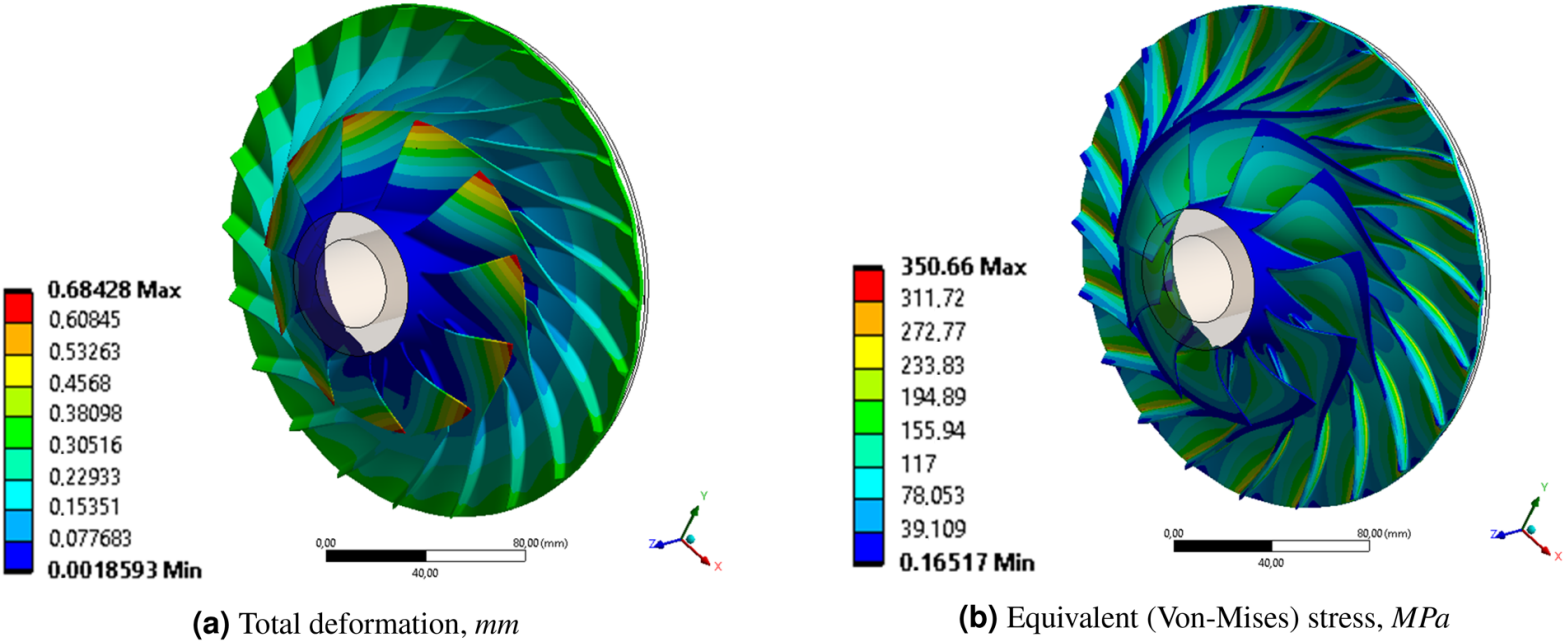
Distribution of (**a**) total deformation and (**b**) equivalent (von Mises) stress of the centrifugal compressor (analysis No. 6) (Ansys Mechanical^34^ visualization).

Depending on the adopted simplification of load modeling, the reduced stresses ranged from 12.1 MPa (analysis No. 5) to 405.41 MPa (analysis No. 1). FSI centrifugation analysis (No. 6, considered the reference result here) gave a value of about 351 MPa (Fig. 5). The location of the highest values of reduced stresses was the place of rounding between the blade and the rotor hub. It follows that the analysis was based on spinning exclusion (No. 1) gives overestimated values of the reduced stresses, while the analysis taking into account only the aerodynamic loads from the flow analysis (No. 5) gives very underestimated values. The closest value of reduced stresses (387.5 MPa) was obtained by taking into account the rotational speed of the rotor (No. 2) and the pressure on the pressure surface of the blades (534.5 kPa). The other two analyses (No. 3 and 4), taking into account the pressure applied on the suction surface of the blade, give values of reduced stresses close to the values from the analysis that take into account only the centrifugation.

**Figure 6:**
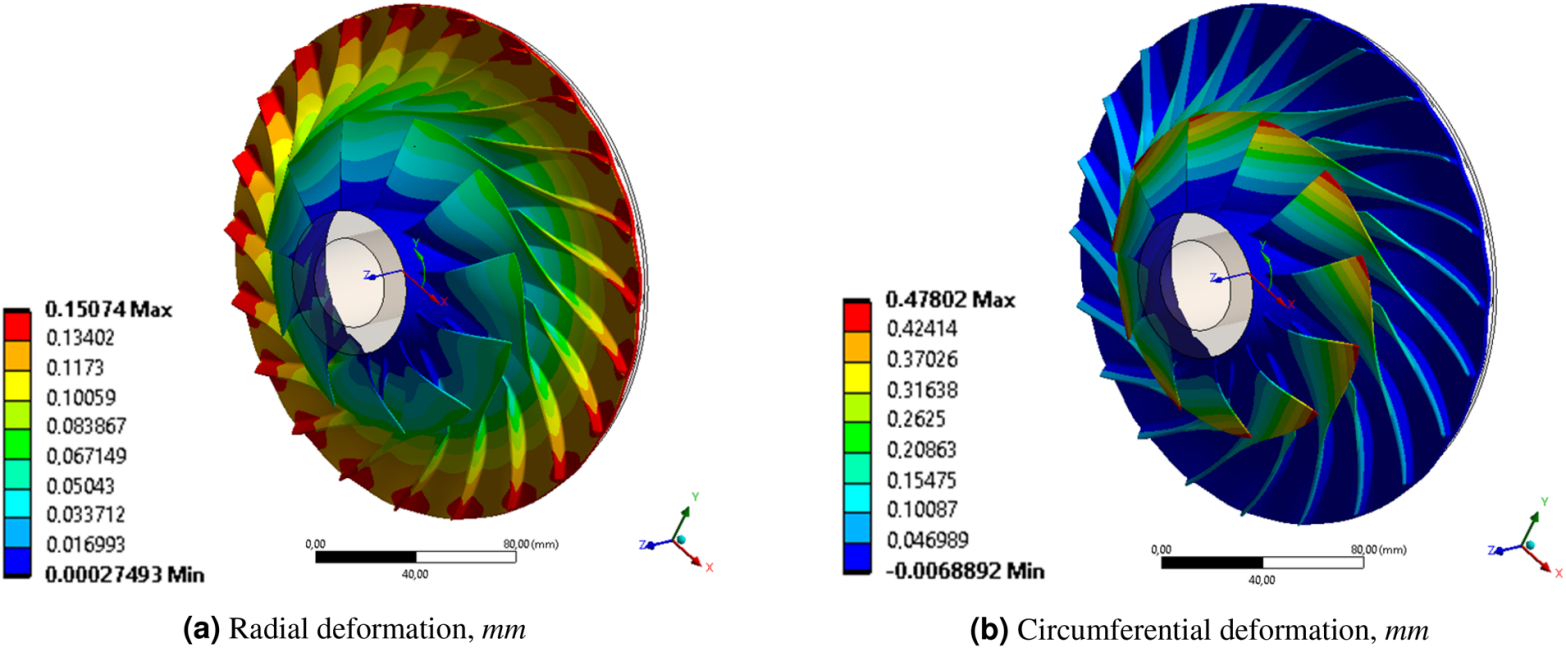
Distribution of (**a**) radial and (**b**) circumferential deformation of the centrifugal compressor (analysis No. 6) (Ansys Mechanical^34^ visualization).

The reference analysis (No. 6) has maximum blade displacements of about 0.684 mm (Fig. 5a), of which 0.151 mm (Fig. 6b) is radial displacement and 0.478 mm (Fig. 6) is circumferential displacement. A positive value of radial displacements, like a positive value of circumferential displacements, implies straightening the blade and bending in the direction opposite to the direction of rotation. The maximum value of total displacements, as well as circumferential displacements, occurs at the inlet to the rotor, at the top of the blade. The maximum values of the radial displacements (Fig. 6) occur at the exit of the rotor. Of all cases of the discussed displacements, analysis No. 1 gives overestimated results (concerning the reference analysis), while the intermediate analysis, taking into account the constant pressure value on the side surfaces of the blades, gives underestimated results.

To determine in detail the distribution of particular types of displacements, as well as the values of the reduced stresses at specific critical points of the compressor rotor, two lines were defined along which the aforementioned values were read and extracted. The first line represents the top of the blade, while the second line represents the rounding in the transition between the blade and the impeller hub. In both cases, the inlet of the defined line starts at the impeller and ends at the impeller end (in the direction of the airflow).

**Figure 7:**
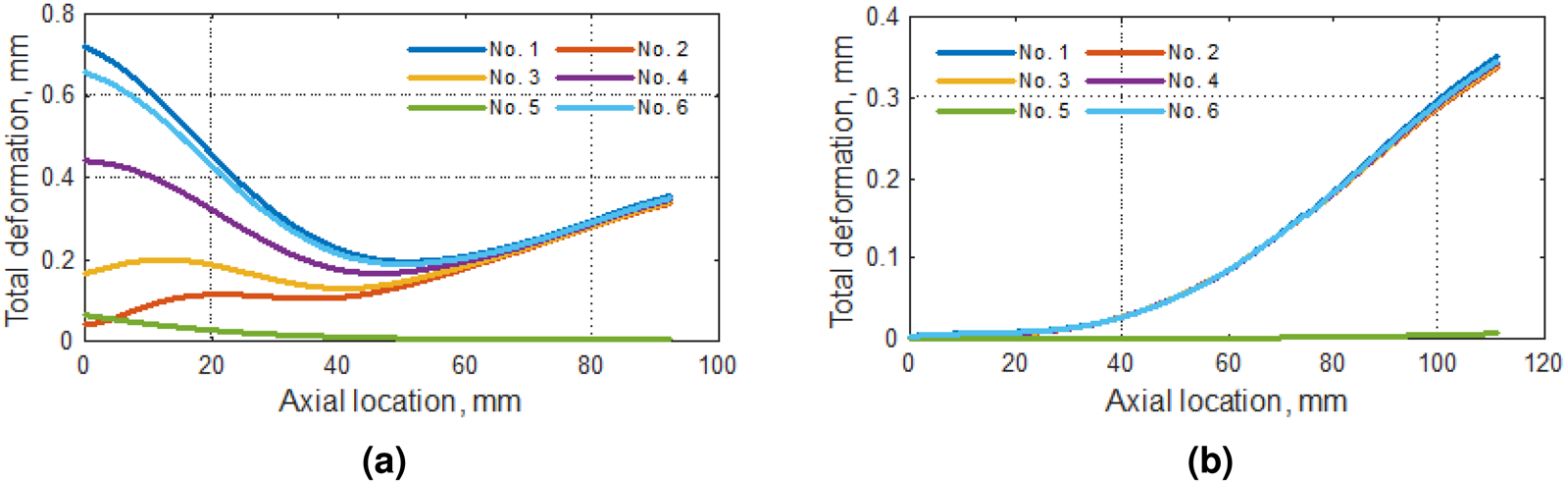
Total deformation along with (**a**) the tip of the blade and (**b**) rounding between the blade and the hub of the compressor.

**Figure 8:**
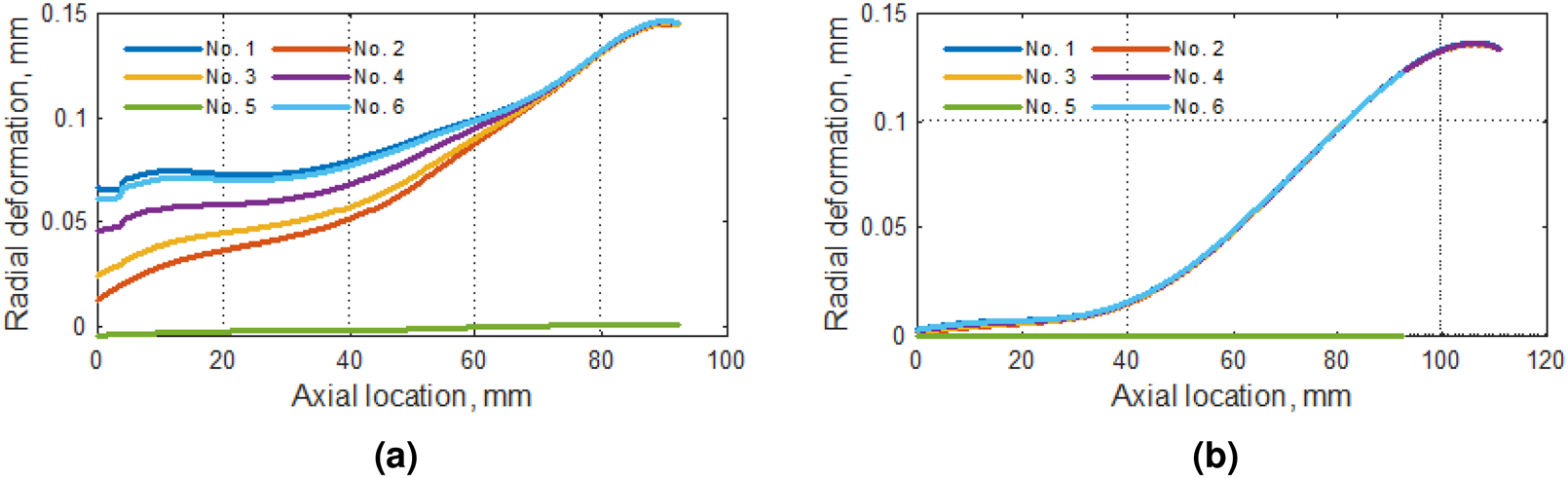
Radial deformation along (**a**) the tip of the blade and (**b**) rounding between the blade and the center of the compressor.

**Figure 9:**
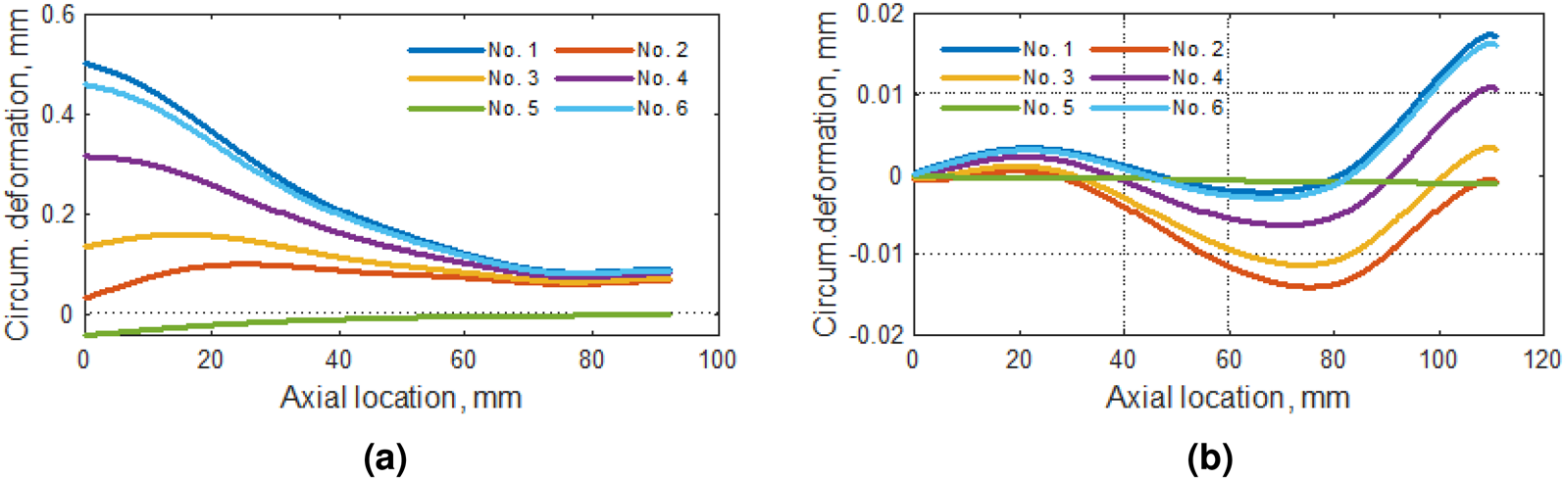
Circumferential deformation along with (**a**) the tip of the blade and (**b**) rounding between the blade and the hub of the compressor.

**Figure 10:**
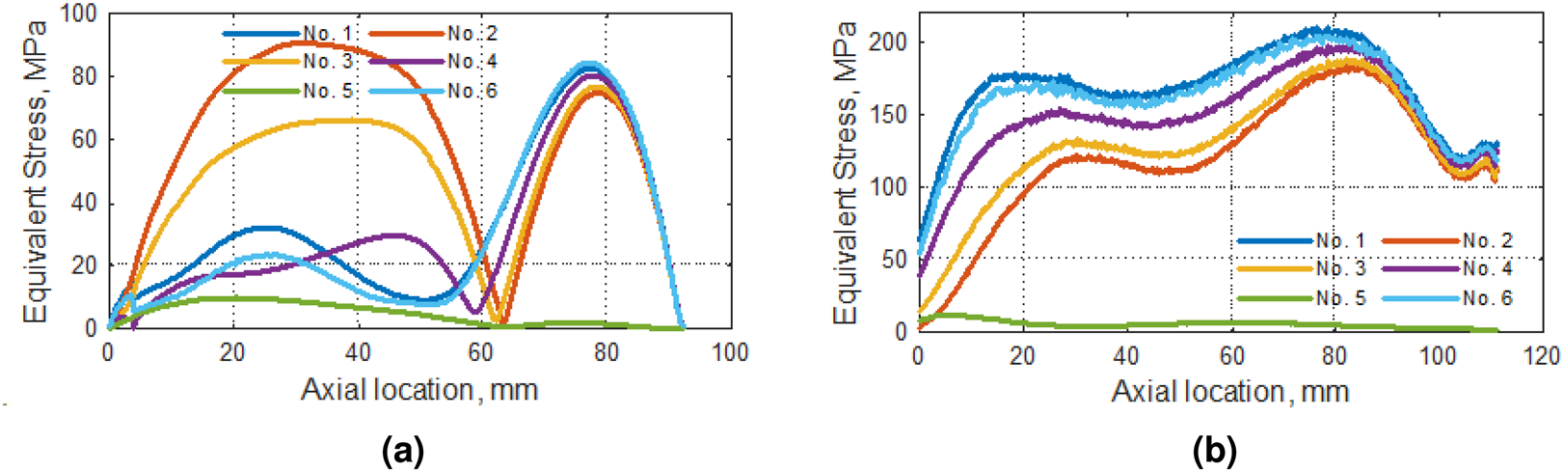
Equivalent stress distribution along (**a**) blade tip and (**b**) rounding between blade and compressor hub.

The read values prepare 4 sets of graphs in Figs. 7, 8, 9 and 10. The results of the analyses carried out were the values of the total (Fig. 7), radial (Fig. 8), and circumferential (Fig. 9) displacements of the blade top and at the rounding between the blade and the rotor hub. Additionally, for the locations mentioned above, the levels of reduced stresses are determined (Fig. 10).

**Table 5:** Results in relative portion to analysis No. 6—FSI with rotation.

Analyze number	Max u$_{\mathrm{tot}}$, mm	Max u$_{\mathrm{cir}}$, mm	Max u$_{\mathrm{rad}}$, mm	Max $\sigma _{eqv}$, MPa
1	1.054	1.052	1	1.156
2	0.529	0.287	0.967	1.105
3	0.531	0.331	0.974	1.116
4	0.645	0.663	0.987	1.136
5	0.0936	0.001	− 0.279	0.035
6	1	1	1	1

In the case of the top of the blade, in line with previous observations, analysis No. 1 gave values over the reference values (No. 6)—Fig. 7a. The remaining analyses gave lower results. In these analyses, one can observe the inflection point of the course, in the vicinity of half of the shoulder blade length. In the case of rounding (Fig. 7b), the total displacements in all cases except analysis No. 5, were the same. The highest value of displacement was observed in the vicinity of the rotor outlet.

Radial displacement analysis showed that for the blade top, analyses No. 1 and 6 give consistent results. In all analyses, the increase in the value of radial displacements was observed with increasing distance from the inlet was observed (except for No. 5). Similar observations were made for the rounding between the blade and the hub (Fig. 8b).

Like the total displacements, the circumferential displacements of the blade tip tend to decrease by about 80% of the blade length (Fig. 9a). In the case of the rounding area (Fig. 9b), the circumferential displacements get very small values (about 100 times smaller than for the blade top).

From the strength point of view, the most important were the results related to the distribution of reduced stresses. It has been observed that at the tip of the blade the stresses were below 100 MPa (Fig. 10a), while in the region of the curvature it was over 200 MPa (Fig. 10b). Research showed that the values of reduced stresses in this part of the rotor were obtained for analysis No. 6. All other analyses gave correspondingly lower results. The closest values were obtained for the analysis No. 1. In the case of the top of the compressor blade, satisfactory results were also obtained in the case of the No. 4, where the working pressure on both sides of the blade was taken into account.

## Comparison

The last step of the work on the obtained results is to determine the error/differences between the analyses concerning the adopted reference value, corresponding to analysis No. 6. These results are summarized in Table 5.

In general, the smallest error in the calculation of displacements (up to 6%) regarding analysis No. 6, obtained thanks to the analysis No. 1. It means that the spinning itself was a good representation of the load status. Unfortunately, these values exceed the values of reduced stresses, which in the case of rotor analysis for aviation applications, may translate into too large a rotor mass. All other load modeling combinations produce underestimated displacement values. Only the radial displacements obtained in analysis No. 4 are satisfactorily similar to the results of Analysis No.6 (error at the level of 1.3%).

In the case of the analysis of the maximum values of reduced stresses, the best result was obtained among all structural analyses (omitting the reference analysis) for analyses 2 and 3. Both analyses assumed working pressure on one (No. 2) or both sides of the blades (No. 3). Analysis No. 2 gives an error of 10%, while analysis No. 1 gives an error of 16%.

Analysis No. 5 was only information on how aerodynamic loads themselves, from flow analysis, affect the load condition on the blade. Modeling loads without considering spin and the associated mass forces was incorrect and should not be considered in future analyses. The results show that aerodynamic loads account for only 10% of the displacement value and about 4.5% of the maximum value of reduced stresses.

## Discussion

The present work demonstrates the impact of modeling aerodynamic loads on the structural deformation and stress state of a centrifugal compressor rotor. The experimental tests carried out on the WESTT test bench made it possible to determine the operating parameters of this rotor, which at the same time became the basis for the numerical flow analysis. CFD analysis allowed us to generate the pressure distribution on the side surfaces of the blades. This approach, combined with the structural analysis, was the basis of one-way fluid-structural analysis, which was considered to be the reference for other analyses.

The modeled numerical studies took into account different ways of applying loads. The analysis took into account centrifugation (inertial forces), operating pressure outer and inner surface of the blade, and aerodynamic forces directly from flow analysis. It was determined that the best indications regarding displacements were obtained in the analyses No. 1 and 4 (error up to 5–6%), while in the case of stress analysis, it was suggested to analyze No. 2 (error of 10%). In general, all the methods presented for modeling aerodynamic loads give conservative results of the maximum values of displacements and stresses that are higher than the reference values.

Based on the analysis conducted, the level of the influence of aerodynamic loads on the total state of the rotor loading is determined. Aerodynamic loads constitute up to 4% of the reduced stresses and up to 10% of the blade structural displacement value.

The results obtained can be used to create a set of rules on which future structural analysis could be based, including simplified methods for modeling aerodynamic loads. In such cases, a series of analyses must be performed considering rotational speeds and different combinations of applied pressures. The developed methodology can be used in the design assessment of centrifugal compressors for aviation, automotive, energy, refrigeration, and other industries that use centrifugal machines.

## Data Availability

All data generated or analysed during this study are included in this published article.
